# Nonlinear pathological trajectory of a high-myopia C57/BL6J mouse model induced by form deprivation

**DOI:** 10.3389/fphys.2024.1442000

**Published:** 2024-10-30

**Authors:** Yue Wen, Yan Li, Li Zhu, Tao Tang, Huichao Yan, Jie Hu, Kai Wang, Mingwei Zhao, Qiong Xu

**Affiliations:** ^1^ Department of Ophthalmology and Clinical Center of Optometry, Peking University People’s Hospital, Beijing, China; ^2^ College of Optometry, Peking University Health Science Center, Beijing, China; ^3^ Eye Disease and Optometry Institute, Peking University People’s Hospital, Beijing, China; ^4^ Beijing Key Laboratory of Diagnosis and Therapy of Retinal and Choroid Diseases, Beijing, China

**Keywords:** form deprivation myopia, high myopia, retinal vessel, choroidal thickness, scleral fiber, nonlinear pathological trajectory

## Abstract

**Introduction:**

To establish a high myopia model in C57BL/6J mice with monocular form deprivation myopia (FDM) and investigate its ocular structure pathological trajectory.

**Methods:**

Healthy 3-week-old C57BL/6J mice were divided into an FDM group (n = 36) and a control group (n = 24). The left eyes of the FDM group were patched, while the right eyes served as controls. Biometric parameters and fundus morphology were assessed at baseline and after 4, 8, and 12 weeks of form deprivation.

**Results:**

Significant differences were observed in the deprived eyes, including longer axial length, higher refractive power, deeper vitreous chambers, thinner retina, choroid, and sclera, and smaller scleral fibers’ diameters under a transmission electron microscope. Retinal vascular area proportion in covered eyes decreased significantly (*P* < 0.05), with a decline rate of 11% from weeks 4 to 8 and a faster decline of 19% from weeks 8 to 12, while this proportion increased significantly in control eyes.

**Discussion:**

This study successfully induced a high myopia model in mice with long-term form deprivation. The axial length grew dramatically in FDM in the first 8 weeks, while the pathological progress of the fundus accelerated from weeks 8 to 12.

## Introduction

Myopia now reaches epidemic proportions. High myopia is characterized as a refraction ≤ −6 D or an axial length (AL) ≥ 26 mm. It is associated with structural changes in the retina, choroid, optic nerve, and sclera (Flitcroft et al., 2019). Pathologic myopia (PM) is characterized as excessive axial elongation caused by myopia, leading to anatomical abnormalities in the posterior segment of the eye, such as posterior staphyloma, myopic chorioretinal atrophy, and retinal detachments, and is a leading cause of blindness worldwide (Ohno-Matsui et al., 2021). It is estimated that nearly half of the world’s population will be myopic by 2050, with up to 10% showing high myopia, and the prevalence of PM in the high myopia population ranges from 50% to 70% (Holden et al., 2016). The incidence rate of myopic maculopathy progression in Chinese children and adolescents was much lower compared with the older population. While AL growth typically decelerates with age, the progression of the fundus remains a concern (Fang et al., 2018; Yan et al., 2018; Guo et al., 2021; Hopf et al., 2022; Foo et al., 2023; Jiang et al., 2024). The current understanding of how these pathological signs evolve from a relatively normal but highly myopic eye remains limited. This knowledge gap underscores the urgency for an in-depth exploration of the pathological progression of high myopia, a condition whose increasing prevalence necessitates a more profound understanding of its trajectory (Morgan et al., 2018). Previous clinical studies have explored the clinical diagnosis, risk factors, and mechanisms of pathologic and high myopia (Morgan et al., 2017; Chen et al., 2018; Shah et al., 2024), yet effective control methods for the progression from high myopia to PM remain elusive. Current control methods are primarily aimed at general myopia progression (Brennan et al., 2024), and treatment options are mainly targeted at complications, such as retinoschisis and posterior staphyloma, through surgical interventions like scleral reinforcement. This limitation is largely due to the unclear mechanisms of the transition from high myopia to PM. Our previous clinical and proteomics studies have sought to identify differentially expressed proteins to uncover related mechanistic pathways (Wen et al., 2024). However, laboratory studies have predominantly focused on myopia mechanisms (Mazade et al., 2024), with scant research on the progression from high to PM and a lack of mature animal models for such studies (Vutipongsatorn et al., 2019). The development of preventive and therapeutic techniques for clinical application is hindered by these knowledge gaps. Our research endeavors to bridge this gap and hopes to contribute modestly to the collective understanding within the field.

The general structure, composition, and pathways of the mouse peripheral retina are very similar to those of humans (Volland et al., 2015), and the reproducibility of refractive and axial length measurements of mice is vital for research (Troilo et al., 2019). Previous myopia induction studies have utilized methods such as monocular form deprivation and negative lens induction, typically for durations of 1–4 weeks (Troilo et al., 2019). By extending the duration of form deprivation, our study aims to emulate the progressive nature of high myopia and to meticulously observe the subsequent alterations in the ocular fundus. It is our hope that through this study, we may identify the mechanisms underlying the transition from high myopia to PM, thereby providing more effective interventions and therapeutic strategies for the prevention or delay of PM in the future.

## Methods

### Animals

We procured sixty healthy, three-week-old C57BL/6J mice from Vital River Laboratory Animal Technology Co., Ltd. (Beijing, China), with an average weight ranging from 10 to 13 g. These mice were subsequently divided into two groups: the form deprivation myopia (FDM) group (n = 36) and the normal control (NC) group (n = 24). As for housing and environmental conditions, during the experimental period, all mice were housed in a controlled environment with a 12-h light/12-h dark cycle. The light phase began at 8:00 AM and was provided by 300-lux white-light LEDs.

### Preparation of the FDM mouse model

**FIGURE 1:**
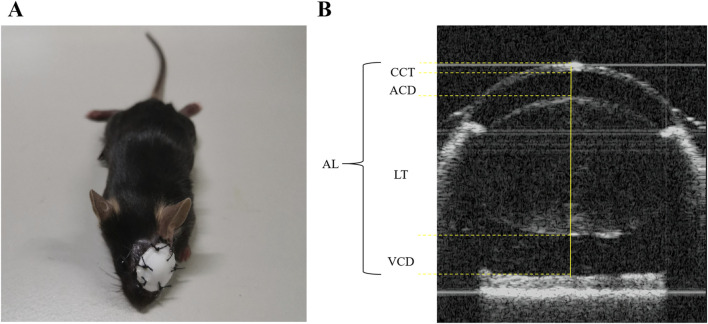
Illustration of the FDM mouse model and biometric measurements. **(A)** The FDM C57BL/6J mouse model. **(B)** The axial length (AL), corneal thickness (CCT), anterior chamber depth (ACD), lens thickness (LT), and vitreous chamber depth (VCD) were measured as shown above.

To induce form deprivation myopia, mice in the FDM group were anesthetized intraperitoneally using 12.5 mg/mL tribromoethanol at a dose of 0.1 mL/10 g. The left eye of each mouse was then sutured with an eye patch (Figure 1A) to deprive it of visual input. The right eye remained untreated, serving as self-control. Conversely, both eyes of the mice in the NC group remained untreated.

To ensure the consistency of the deprivation, the eye patches were inspected daily, and any patches that fell off were promptly reapplied. The size of the eye patches was adjusted accordingly to match the growing mice throughout the experiment. The induction process was maintained for a duration of 12 weeks.

### Biometric measurements

Prior to the induction of myopia, biometric assessments encompassing axial length (AL), refraction, and additional ocular parameters were conducted in both the FDM and NC groups. These evaluations were repeated at weeks 4, 8, and 12 post-induction. Following intraperitoneal anesthesia, the ocular patches were delicately removed with scissors, and 5 mg/mL of tropicamide phenylephrine eye drops (Santen Pharmaceutical Co., Ltd. Shiga Plant) were administered to the mice’s eyes. Refraction was gauged using a photorefractor (Striatech, Germany), while an optical coherence tomography scanner specifically designed for animals was employed to measure AL, central corneal thickness (CCT), anterior chamber depth (ACD), lens thickness (LT), and vitreous chamber depth (VCD). To ensure accuracy, each eye underwent at least 50 refractive examinations and three AL measurements, and the average of these readings was taken as the final result. The methodology for measuring AL, CCT, ACD, LT, and VCD is illustrated in Figure 1B.

### Retinal thickness (RT) and choroidal thickness (ChT) measurements

**FIGURE 2:**
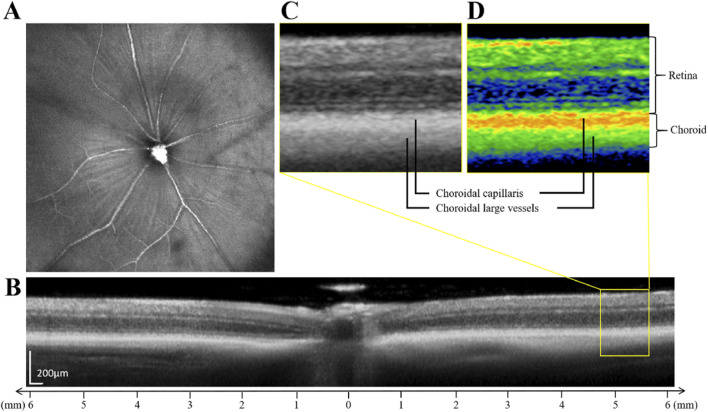
Illustration of the retinal thickness and choroidal thickness measurements. **(A)** SS-OCT fundus imaging of mice. **(B)** The schematic diagram of retinal and choroidal layering. **(C)** Enlarged original image in the yellow box in figure **(B)**. **(D)** Enlarged pseudo-color image in the yellow box in figure **(B)**. The choroidal layer is further divided into the choroidal capillaris and the choroidal large vessels.

To precisely quantify the retinal thickness (RT) and choroidal thickness (ChT) in our experimental mice, we employed a swept-source optical coherence tomography (SS-OCT) system (TowardPi Medical Technology, Beijing, China). The mice were scanned at the center of their optic discs as shown in Figure 2A, and the retinal and choroidal layers were delineated as depicted in Figures 2B–D. Specifically, the measurements were taken horizontally, on both the nasal and temporal sides, spanning 3–6 mm away from the optic nerve. This approach allowed us to capture the variations in RT and ChT across these critical regions.

### Color fundus photography and fluorescein fundus angiography

For fundus photography and angiography, the mice were initially fixed on an elevated table under anesthesia. The pupils were dilated using 5 mg/mL tropicamide phenylephrine eye drops, and surface anesthesia was achieved with 5 mg/mL proparacaine hydrochloride eye drops. Once the pupils were adequately dilated and the eye position stabilized, carbomer eye drops were applied to the cornea of the eye to be examined. Subsequently, the microscope lens of the fundus imaging system designed for small animals was gently brought into contact with the eye, and color fundus photographs were captured. To investigate the pathological vessel features of form-deprivation-induced high myopia in a mouse model, we followed an angiography experimental protocol. Initially, the light source was shifted to the Blue Channel, and the light intensity was fine-tuned. The manual filter wheel on the camera was adjusted accordingly. Subsequently, an intraperitoneal injection of 2% fluorescein sodium (1.7 mL/kg) was administered to the mice. Following this, the experimental table and lens focus were precisely calibrated to capture photographs of both eyes of the mice. Post-experiment, both eyes were thoroughly rinsed with saline, and levofloxacin eye drops were administered to mitigate the risk of infection.

### Histopathological staining and transmission electron microscopy (TEM)

For histopathological analysis and transmission electron microscopy (TEM), the mice were anesthetized at designated time points of weeks 4, 8, and 12. Their eyes were carefully extracted and immediately immersed in FAS Eyeball Fixative (Servicebio, Wuhan, China) for fixation. Subsequently, the eyeballs underwent paraffin embedding, followed by sectioning along the optic nerve. Hematoxylin-eosin (H&E) staining and dehydration sealing were performed to prepare the samples for microscopic examination. The stained sections were observed and imaged under a light microscope (Olympus, Japan) at various magnifications (4x, 10x, 20x, and 40x). Furthermore, scleral thickness was measured three times at a distance of 500 μm from the optic nerve to ensure consistency and accuracy. For the TEM analysis of the eyeballs, the following protocol was adhered to. After removing the eyeballs, within 1–3 min, we isolated a 2 mm × 2 mm section comprising the entire layers of retinal, choroidal, and scleral tissues. These tissue sections were promptly transferred into an EP tube containing fresh fixative (Servicebio, Wuhan, China) specific for TEM. The tissues were fixed for 2 h at room temperature and then stored at 4°C.

Subsequently, the tissue sections were washed thoroughly using 0.1 M phosphate buffer (pH 7.4), repeating the process three times, with each wash lasting for 15 min. Following the washes, the tissues underwent dehydration at room temperature, resin penetration, and embedding. Once embedded, the samples were polymerized and ultrathin sections were prepared. These sections were then stained using appropriate methods.

### Statistical analysis

IBM Statistics SPSS 24 and GraphPad Prism 10.0 software were applied for statistical analysis. Descriptive statistics characterized participant demographics. Normality was tested using Shapiro-Wilk, and variance homogeneity was confirmed. One-way ANOVA (for homoscedasticity) and Welch ANOVA (for heteroscedasticity) compared ocular measures like AL, CCT, ACD, LT, VCD, VCD/AL ratio, refraction, RT, ChT, and retinal vascular area percentage between FDM and NC groups over time, with Bonferroni adjustments for multiple comparisons. Paired-samples *t*-tests analyzed FDM group parameter changes over time. Measurements of AL, CCT, ACD, LT, VCD, and scleral fibers’ Feret’s diameters were accurately obtained using ImageJ software. Furthermore, the distribution of scleral fibers’ diameters and cumulative frequency curves were plotted using Origin 2021 software. For retinal vascular quantitative analysis, Angiotool software was employed to assess vascular parameters (Zudaire et al., 2011). Two-sided tests were performed, and *P* < 0.05 was considered statistically significant.

## Results

### Variation of biometric parameters

**FIGURE 3:**
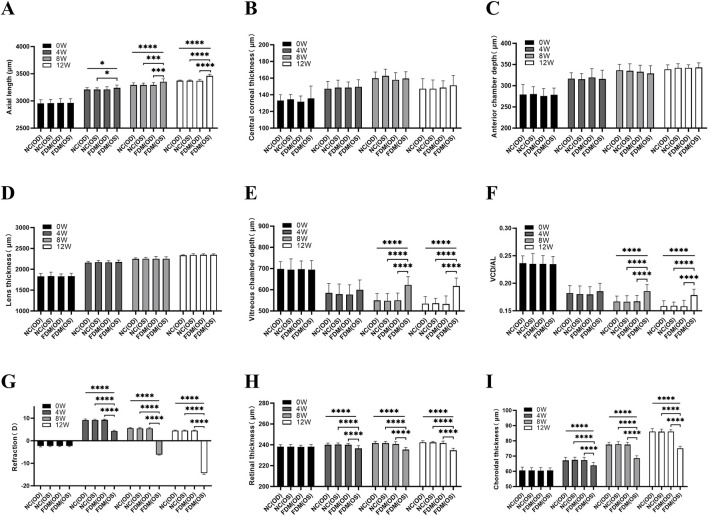
Biometric measurements. **(A)** Axial length (AL). **(B)** Central corneal thickness (CCT). **(C)** Anterior chamber depth (ACD). **(D)** Lens thickness (LT). **(E)** Vitreous chamber depth (VCD). **(F)** VCD/AL, the ratio of VCD to AL. **(G)** Refraction. **(H)** Retinal thickness (RT). **(I)** Choroidal thickness (ChT). NC (OD) is the right eye of the normal control (NC) group, NC (OS) is the left eye of the NC group, FDM (OD) is the right eye of the form deprivation myopia (FDM) group, and FDM (OS) is the left (covered) eye of the FDM group. The FDM group was compared with the NC group. At 0, 4, 8, and 12 weeks, the AL, CCT, ACD, LT, VCD, VCD/AL, refraction, RT, and ChT in the right and left eyes of mice were compared between the FDM and NC group. One-way ANOVA (for homoscedastic) and Welch ANOVA (for heteroscedastic) test were used followed by Bonferroni adjustments for multiple comparisons. ^*^ *P* < 0.05, ^***^ *P* < 0.001, ^****^ *P* < 0.0001.

As shown in Figure 3 and Supplementary Table S1, prior to the induction of myopia, our evaluation of the AL, VCD, VCD/AL, and refraction in both eyes of the mice revealed no significant differences between the FDM and NC groups (all *P* > 0.05, one-way ANOVA). However, upon inducing myopia for durations of 4, 8, and 12 weeks, we observed marked increases in the AL of the covered (left) eyes in the FDM group, as compared to the self-control (right) eyes within the FDM group and the ipsilateral (left) eyes in the NC group (Figure 3A, *P < 0.05, ***P < 0.001, ****P < 0.0001, one-way ANOVA). At 8 and 12 weeks post-induction, we observed marked increases in the VCD and VCD/AL for the left eyes in the FDM group when compared to the right eyes within the FDM group and the left eyes in the NC group (Figures 3E, F, ****P < 0.0001, one-way ANOVA). At each time point, there were no significant differences in CCT, ACD, or LT between the eyes of two groups (Figures 3B–D, all *P* > 0.05, one-way ANOVA). Furthermore, the refraction of the left eyes in the FDM group was lower than those of the right eyes within the FDM group and the left eyes in the NC group (Figure 3G, ****P < 0.0001, one-way ANOVA and Welch ANOVA).

### Variation of the RT and ChT

Utilizing spectral-domain optical coherence tomography (SS-OCT), we measured the RT and ChT of the mice. Our initial findings, prior to myopia induction, indicated no significant variations of RT and ChT between the two groups (all *P* > 0.05, one-way ANOVA). However, following the 4, 8, and 12-week induction period, a substantial reduction in both RT and ChT was evident in the covered (left) eyes of the FDM group (Figures 3H, I, ****P < 0.0001, one-way ANOVA).

### Color fundus photography and fluorescein fundus angiography

**FIGURE 4:**
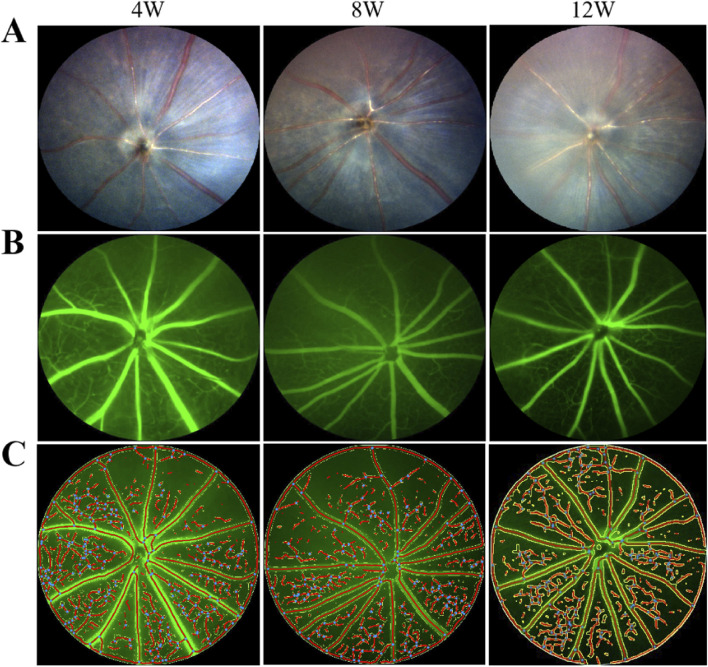
Fundus photography and fluorescein angiography of covered (left) eyes of mice in the FDM group. **(A)** From left to right are fundus photographs at weeks 4, 8, and 12. **(B)** From left to right are fundus fluorescein angiograms at weeks 4, 8, and 12. **(C)** From left to right are schematic diagrams of quantitative analysis of the retinal vessels at weeks 4, 8, and 12. With the prolongation of induction time, the retinal vascular area ratio decreased in the left eyes of mice in the FDM group. *F* = 40.97, *P* = 0.0003, one-way ANOVA.

As depicted in Figure 4, with the progressive extension of the induction period, the percentage of retinal vascular area in the covered (left) eyes (37.30% ± 0.83% at week 4, 33.16% ± 1.07% at week 8, 26.82% ± 2.07% at week 12) of the mice in the FDM group exhibited a notable decrease (*F* = 40.97, *P* = 0.0003), and the percentages of retinal vascular area of the left eyes at weeks 8 and 12 were significantly lower than those of the self-control (right) eyes (*t* = −20.25, *P* = 0.0024 at week 8; *t* = −9.541, *P* = 0.0108 at week 12). Specifically, the rate of decrease was 11% from weeks 4–8 and further escalated to 19% from weeks 8–12. In contrast, the retinal vascular area of the right eyes (37.61% ± 1.18% at week 4, 42.56% ± 1.58% at week 8, 47.00% ± 1.66% at week 12) showed an increase over the same period (*F* = 29.97, *P* = 0.0008).

### Histopathological staining

**FIGURE 5:**
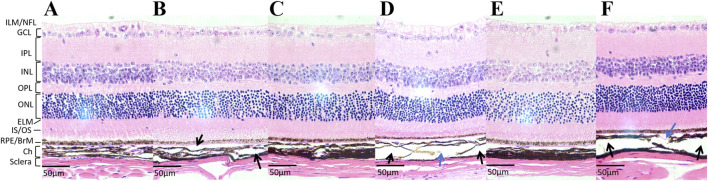
The hematoxylin-eosin (H&E) staining images of tissue sections at a 40x microscope in the FMD group. **(A)** The tissue section of the self-control (right) eye at week 4. **(B)** The tissue section of the covered (left) eye at week 4. **(C)** The tissue section of the right eye at week 8. **(D)** The tissue section of the left eye at week 8. **(E)** The tissue section of the right eye at week 12. **(F)** The tissue section of the left eye at week 12. Compared with the right eye, the left eye exhibited a thinning of the inner nuclear layer, outer nuclear layer, and retinal pigment epithelium. Additionally, the choroidal vascular layer appeared sparse, accompanied by significant tissue degradation (sparse layers marked by black arrows and tissue degradation indicated by blue arrows in Figures 5B, D, F).

**FIGURE 6:**
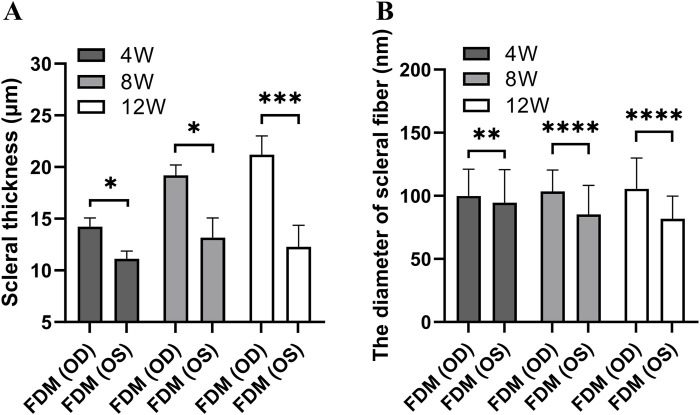
The scleral thickness and diameters of scleral fibers in the FDM group. **(A)** The scleral thickness. **(B)** The diameters of scleral fibers. A paired-samples *t*-test was used to compare the scleral thickness and diameters of scleral fibers of eyes in the FDM group at different time points. The scleral thicknesses of the left eyes at weeks 4, 8, and 12 were significantly thinner than those of the right eyes (*t* = −8.440, *P* = 0.0138; *t* = −9.490, *P* = 0.0109; *t* = −52.292, *P* = 0.0004). The scleral fibers’ diameters of the left eyes in the FDM group were thinner than those of the right eyes at weeks 4, 8, and 12 (*t* = −3.24, *P* = 0.0012; *t* = −13.72, *P* < 0.0001; *t* = −14.61, *P* < 0.0001).

Light microscopic examination of the retinal, choroidal, and scleral structures in mice from the FDM group at 40x magnification at weeks 4, 8, and 12 revealed distinct changes (Figure 5). Compared with the self-control (right) eye, the covered (left) eye exhibited a thinning of the inner nuclear layer, outer nuclear layer, and retinal pigment epithelium. Additionally, the choroidal vascular layer appeared sparse, accompanied by significant tissue degradation (sparse layers marked by black arrows and tissue degradation indicated by blue arrows in Figures 5B, D, F). Notably, the scleral thicknesses of the left eyes at weeks 4, 8, and 12 (11.12 ± 0.75 μm at week 4, 13.18 ± 1.89 μm at week 8, 12.29 ± 2.09 μm at week 12) were significantly thinner than those of the right eyes (14.23 ± 0.84 μm at week 4, 19.20 ± 1.00 μm at week 8, 21.19 ± 1.81 μm at week 12). As shown in Figure 6A, these differences were statistically significant (*t* = −8.440, *P* = 0.0138 at week 4; *t* = −9.490, *p* = 0.0109 at week 8; *t* = −52.292, *P* = 0.0004 at week 12).

### Transmission electron microscopy (TEM)

**FIGURE 7:**
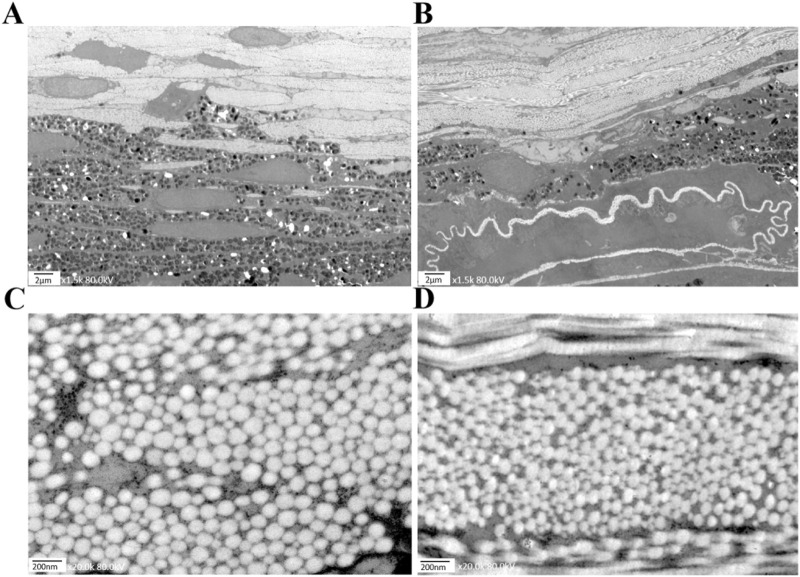
Electron micrographs of mice in the FDM group at week 12. **(A)** The choroid structure in the self-control (right) eye at 1.5 kx under TEM. **(B)** The choroid structure in the covered (left) eye at 1.5 kx under TEM was thinner than that of the right eye, with fewer vascular lumens and disorganized structures. **(C)** The scleral fibers in the right eye at 20 kx under TEM. **(D)** The scleral fibers in the left eye at 20 kx under TEM.

Utilizing Transmission Electron Microscopy (TEM), we observed distinct pathological changes in the choroid and sclera of the covered (left) eyes of mice subjected to form deprivation myopia (FDM) compared to their self-control (right) eyes. Specifically, at week 12, the choroid thickness in the left eyes (Figure 7B) was significantly reduced, accompanied by a decrease in vascular lumens and an increase in the presence of disorganized structural arrangements, in contrast to the right eyes (Figure 7A).

**FIGURE 8:**
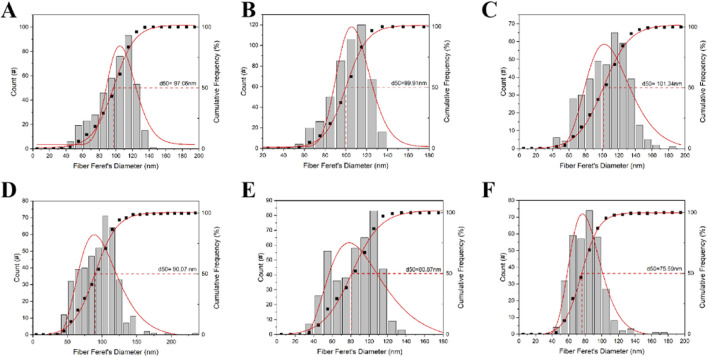
The distributions and cumulative frequency curves of scleral fiber diameters of mice in the FDM group. **(A)** Scleral fibers’ diameters’ distribution and cumulative frequency curve of the self-control eye (right) at week 4. **(B)** Scleral fibers’ diameters’ distribution and cumulative frequency curve of the right eye at week 8. **(C)** Scleral fibers’ diameters’ distribution and cumulative frequency curve of the right eye at week 12. **(D)** Scleral fibers’ diameter distribution and cumulative frequency curve of the occluded (left) eye at week 4. **(E)** Scleral fibers’ diameters’ distribution and cumulative frequency curve of the left eye at week 8. **(F)** Scleral fibers’ diameters’ distribution and cumulative frequency curve of the left eye at week 12. Scleral fibers’ diameters’ at 50% of the cumulative frequency curve (d50) was also found to be smaller in the left eyes compared to the right eyes in the FDM group. With increasing induction time, scleral fiber diameters decreased in the left eye (*F* = 31.85, *P* < 0.0001) and increased in the right eye (*F* = 7.68, *P* = 0.0005).

A similar trend was observed in the scleral fibers, where the diameters in the left eyes (Figure 7D) were notably thinner than those in the right eyes (Figure 7C). As shown in Figure 6B, our findings clearly demonstrate that the scleral fibers’ diameters of the left eyes in the FDM group were consistently smaller compared to those of the right eyes (94.59 ± 26.22 nm vs. 99.93 ± 21.08 nm at week 4, 85.26 ± 22.98 nm vs. 103.41 ± 17.03 nm at week 8, 81.78 ± 18.00 nm vs. 105.57 ± 24.33 nm at week 12). These differences were statistically significant (*t* = −3.24, *P* = 0.0012 at week 4; *t* = −13.72, *P* < 0.0001 at week 8; *t* = −14.61, *P* < 0.0001 at week 12). To further quantify these changes, we analyzed the distributions and cumulative frequency curves of scleral fibers’ diameters at 20 kx magnification under TEM (Figure 8). Notably, the scleral fiber’s diameter at 50% of the cumulative frequency curve (d50) was also found to be smaller in the left eyes compared to the right eyes. Additionally, with the progression of the induction period, a decreasing trend in scleral fibers’ diameters was observed in the left eyes (*F* = 31.85, *P* < 0.0001), while an increasing trend was noted in the right eyes (*F* = 7.68, *P* = 0.0005).

## Discussion

In this study, we successfully established a form deprivation myopia (FDM) model in C57BL/6J mice to investigate the pathological characteristics of high myopia. Our findings revealed significant differences in ocular parameters between the FDM group and the NC group. In the NC group, we observed a gradual increase in AL, ACD, and LT, accompanied by a decrease in VCD with age. However, in the FDM group, the AL growth of the covered eyes was significantly accelerated compared to the control eyes. Covered eyes showed a significant reduction in VCD at week 4, but then exhibited an increasing trend over time. The refraction of the covered eye in the FDM group ultimately reached −14.37 D, with an 18.83D difference from the self-control eye, and an 85.05 μm difference in VCD between the eyes was observed. By extending our observation period to 12 weeks, we noted a significant increase in refractive error compared to previous studies with shorter deprivation durations (Tkatchenko et al., 2010a; Zi et al., 2020; Zhu et al., 2021). Furthermore, the retinal thickness, choroidal thickness, and posterior scleral thickness were all significantly reduced in the covered eyes, and a progressive decrease in the proportion of retinal blood vessels was observed. Histological analysis using H&E staining revealed sparse and disorganized posterior choroidal structures in the FDM group. Additionally, TEM revealed a decrease in the diameter of scleral fibers following myopic induction. These findings are particularly noteworthy as chorioretinal atrophy and scleral thinning at the posterior pole are crucial pathological features in the progression of high myopia in humans, ultimately leading to the formation of posterior scleral staphylomas (McBrien, 2013; Wu et al., 2018).

Previous research has established animal models of simple axial myopia in various species, including chicks (Biswas et al., 2023), mice (Zhu et al., 2021; Wu et al., 2022), guinea pigs (Zhao et al., 2021; Zhang et al., 2023), rabbits (Rong et al., 2017), and tree shrews (McBrien, 2013). Although fundus lacquer cracks have been observed in chicks following eyelid suture closure, chorioretinal atrophy has not been consistently reported (Hirata and Negi, 1998). In recent years, several investigators have reported the occurrence of early lacquer cracks in the guinea pig model of spontaneous myopia (Zhang et al., 2023). Similarly, pathological myopic fundus changes have been documented in animal models harboring specific gene mutations. However, it is noteworthy that myopia is often a complex phenotype, resulting from the interplay of multiple genetic abnormalities and environmental factors (Zhao et al., 2020). Therefore, animals with single gene mutations may not fully recapitulate the complex etiology of myopia in humans, limiting their usefulness as research models for the widespread progression mechanisms of high myopia.

Given the availability of C57BL/6J mice, which possess a well-defined genetic background and an extensive array of relevant testing reagents, these animals offer a more suitable alternative for studying myopia (Brown et al., 2022). Moreover, the ocular structure and developmental processes in C57BL/6J mice are more analogous to those in humans, making them a favorable choice for investigating the molecular mechanisms underlying the onset and progression of PM. Thus, we opted to use C57BL/6J mice as our research model in our current study. In the previous study using a normal C57BL/6J mouse, the eyes continued to develop even after 89 days of birth, with the period of highest susceptibility for experimental myopia occurring within the first 67 days after birth (Tkatchenko et al., 2010a; Tkatchenko et al., 2010b). The mice often started with myopic refractive error but then progressed to more hyperopic refractive errors with age and reached a stable refraction of hyperopia. Besides, VCD decreased from 0.751 mm at day 22 to 0.650 mm by day 47, owing to significant lens enlargement, and slightly reduced further to 0.603 mm by day 102. Notably, in normally developing mice, the LT/AL ratio exceeds 60%, and the LT increases substantially during development, causing VCD to decline as the enlarging lens occupies the available space (Zhou et al., 2008; Chakraborty et al., 2017). In contrast, humans do not exhibit such a high LT/AL ratio or rapid lens growth encroaching on the vitreous chamber. Human VCD gradually increases with age, significantly contributing to AL elongation, particularly in highly myopic individuals where the remodeling of the sclera and rapid VCD growth are significant risk factors for axial elongation (Takkar et al., 2019; Paritala et al., 2022).

This study observed a progressive increase in LT from baseline to 12 weeks post-induction in the eyes, yet no significant difference was noted between covered eyes and control eyes (Figure 3D). Concurrently, VCD in control eyes exhibited a gradual decline over the same period, consistent with previous studies (Zhou et al., 2008). In contrast, covered eyes showed a significant reduction in VCD at week 4, but then exhibited an increasing trend over time. Notably, the VCD of covered eyes at weeks 8 and 12 was longer than that at week 4 and significantly longer than those of control eyes. The difference in VCD between covered eyes and control eyes consistently increased over time. Interestingly, in the 12-week FDM model in mice, a decrease in VCD from baseline was observed due to lens thickening. However, an increase in VCD was also observed during the progression of myopia, mirroring the changes seen in human myopia progression. Therefore, although mice have their own unique ocular structural characteristics, with VCD being compressed by lens thickening and decreasing over time, the increase in VCD in the myopia model leads to axial elongation in mice, similar to humans, making it a valuable animal model for studying human myopia.

The extension of the induction period to 12 weeks in our study provided a more comprehensive understanding of the developmental trajectory of myopia in the FDM mouse model. The rapid increase in AL and VCD during the initial 8 weeks of induction underscores the sensitivity of the ocular system to environmental stimuli during this critical period of development. The deceleration in the growth rate from weeks 8–12, despite the continued increase in AL and VCD, provides an intriguing insight into the nonlinear dynamics of myopia progression. This transition in the growth pattern suggests that, although the initial deprivation stimulus triggers a rapid elongation of the eye, over time, the ocular system may begin to exhibit compensatory mechanisms that slow down this process.

A noteworthy observation in our study is the accelerated decline in the proportion of retinal blood vessels in the covered eyes of mice during the latter phase of the induction period. Specifically, the rate of decrease was 11% from weeks 4–8, which escalated to 19% from weeks 8–12. This acceleration in the rate of vascular regression, despite a slowdown in the axial length growth of the covered eyes from weeks 8–12, suggests a dissociation between the two processes. It is intriguing that while the ocular elongation appears to be plateauing, the progression of fundus changes, including the vascular regression, is actually accelerating. The finding that the proportion of retinal blood vessels in the control eyes increased with age is an interesting observation that has not been reported in prior studies, which is contrary to the trend observed in the covered eyes and further underscores the specificity of the changes induced by FDM. The decrease in vascularization, along with the thinning of the retina and choroid, reduction in vessel lumen, thinning of sclera, and reduction in scleral fibers’ diameters observed in histological and ultrastructural analyses, suggests a progressive degradation of ocular tissues with increasing induction time. These findings provide valuable insights into the pathogenesis of myopia and potential targets for therapeutic interventions.

Our results are corroborated by clinical observations that pediatric and adolescent myopia can progress at a rate of up to 0.2–0.3 mm/year, while in adults with high myopia, the AL increase is generally no higher than 0.11 mm per year. However, severe complications predominantly manifest after the age of 40 (Saka et al., 2010; Kong et al., 2024). Similar to our murine model, human studies have shown that while AL growth typically slows with age, fundus changes may continue to progress (Chen et al., 2018). Notably, older individuals with elongated ALs are more prone to severe myopia complications than their younger counterparts (Ducloux et al., 2023). It is imperative to closely monitor the AL and fundus of highly myopic participants during their young adulthood, even without fundus lesions. Existing therapeutic strategies for high myopia include conservative and surgical treatments, with posterior scleral reinforcement being the only surgical method aimed at curbing the progression (Huang et al., 2019). However, this intervention targets the symptom rather than the underlying cause and poses a risk of serious complications, primarily due to our limited understanding of the mechanisms driving the transition from high to PM. In this context, our mouse model is ideal for studying drugs and optical treatments to prevent high myopia from progressing to PM. In the future, we hope to develop more targeted and effective treatments for high myopia by translating these findings into clinical practice.

One of the limitations of our current study is the challenge of extending the duration of form deprivation. When we attempted to extend the induction period beyond 12 weeks, we encountered issues with the eye patches being occasionally dislodged by the mice. These complications not only limited the duration of monocular form deprivation but also potentially hindered our ability to observe the long-term pathological changes in the fundus of the eyes with extended deprivation periods. Our ongoing research, which involves the use of a 3D-printed neck collar to reinforce the eye patch, aims to explore potential pathological changes associated with myopia under extended periods of form deprivation. A single instance of corneal opacity due to infection was encountered in one mouse, impacting the experiment’s continuation. This case was meticulously excluded from our analysis without compromising the sample size’s integrity. Subsequently, we have adopted a protocol of increased frequency in administering topical antibiotic eyedrops and have intensified our cleaning procedures, which has resulted in no further occurrences of such complications.

In conclusion, our study has successfully developed a mouse model of high myopia induced by long-term form deprivation, demonstrating robust operability and reproducibility. This model serves as an invaluable resource for elucidating the mechanisms that drive the transition from high myopia to pathologic myopia. By delving into the underlying mechanisms, we aim to identify key signaling pathways or optical environment modifications that could halt or reverse the progression from high to pathologic myopia. These findings are crucial for advancing our understanding of the disease and potential therapeutic strategies. In the future, we hope to develop more targeted and effective treatments for high myopia by translating these findings into clinical practice.

## Data Availability

The raw data supporting the conclusions of this article will be made available by the authors, without undue reservation.
